# Kaolinite Thin Films Grown by Pulsed Laser Deposition and Matrix Assisted Pulsed Laser Evaporation

**DOI:** 10.3390/nano12030546

**Published:** 2022-02-05

**Authors:** Luminita Nicoleta Dumitrescu, Eusebiu-Rosini Ionita, Ruxandra Birjega, Andrada Lazea-Stoyanova, Maria-Daniela Ionita, George Epurescu, Ana-Maria Banici, Simona Brajnicov, Florin Andrei, Andreea Matei

**Affiliations:** 1 Lasers Department, National Institute for Lasers, Plasma and Radiation Physics, 409 Atomistilor Street, 077125 Magurele, Romania; nicoleta.dumitrescu@inflpr.ro (L.N.D.); ruxandra.birjega@inflpr.ro (R.B.); george.epurescu@inflpr.ro (G.E.); ana.niculescu@inflpr.ro (A.-M.B.); brajnicov.simona@inflpr.ro (S.B.); andreea.matei@inflpr.ro (A.M.); 2 Sources for Plasma and Applications Group, Low Temperature Plasma Laboratory, National Institute for Lasers, Plasma and Radiation Physics, 409 Atomistilor Street, 077125 Magurele, Romania; andrada@infim.ro (A.L.-S.); daniela.ionita@infim.ro (M.-D.I.); 3 Innovation Center in Photonics and Plasma for Advanced Materials and Technologies FOTOPLASMAT, National Institute for Lasers, Plasma and Radiation Physics, 409 Atomistilor Street, 077125 Magurele, Romania; florin.andrei@inflpr.ro

**Keywords:** laser processing, kaolinite, lamellar materials, thin films

## Abstract

In this work, thin films of lamellar clays were deposited by laser techniques (matrix assisted pulsed laser evaporation (MAPLE) and pulsed laser deposition (PLD)). The focus of this paper is the optimization of deposition parameters for the production of highly oriented crystalline films. The films were characterized by X-ray diffraction (XRD), X-ray photoelectron spectroscopy (XPS), atomic force microscopy (AFM), and scanning electron microscopy (SEM). Contact angle measurements were employed to identify the wetting properties of the deposited thin films. Hydrophobic to superhydrophilic films can be prepared by using different deposition techniques and deposition parameters. MAPLE led to superhydrophilic films with contact angles in the range 4°–8°, depending on the microstructure and surface roughness at micro and nano scale. The 1064 nm PLD had a high deposition rate and produced a textured film while at λ = 193 nm an extremely thin and amorphous layer was depicted. Oriented kaolinite films were obtained by MAPLE even at 5 wt.% kaolinite in the target.

## 1. Introduction

Lamellar clay materials are promising candidates as low-cost adsorbents with protein-binding capacities. Environmental industry uses clay minerals intensively for a variety of applications ranging from absorbance and storage of hazardous chemicals up to cleaning of polluted waters and sensing elements [1]. The main property of these lamellar materials is the adsorption capability, which is connected to the layer charge density, cationic charge capacity and swelling characteristic [2]. Kaolinite, being the most common clay mineral, is a lamellar material with the chemical formula (Al_2_O_3_ · 2SiO_2_ · 2H_2_O) and is used in several important fields as paper fabrication, pharmaceutical industry, cosmetics, or as adsorbents in water and wastewater treatment and many more [3]. These applications are based on the lamellar guest-host structure, which can adsorb a wide range of molecules, at the surface, at the edge of the lamellas or in the inter-lamellas [4]. Grafting reactions have been studied intensively in the last decades. For example, modified electrodes for electrochemical applications were synthesized in a two steps procedure, first grafting triethanolamine on the interlayer aluminol groups and then reacting with iodomethane for cyanide anions detection [5]. Raw clays treated with hydrochloric acid and acetic acid were tested for adsorption of Cr (VI) and Fe (III) [6] and kaolin/polymer nanocomposite membranes were prepared for the removal of arsenic from aqueous solutions [7]. The detection of iodide in aqueous solution containing thiosulfate was reported onto a gold electrode modified by a thin film of kaolinite, obtained by grafting the ionic liquid (1-(2-hydroxyethyl)-4-(tert-butyl) pyridinium chloride) [8]. Wettability of hydrogen on kaolinite clay surfaces, in the presence of brine, was investigated for further use in hydrogen storage, with improved results as compared to illite or montmorillonite [9].Functionalized kaolinite hybrid materials with controlled interlayer distance were obtained by grafting trihydroxyethylmethylammonium iodide, 1-(2-hydroxyethyl)-3-methylimidazolium chloride and 1-benzyl-3-(2-hydroxyethyl) imidazolium chloride and were used as modified electrodes for electrochemical detection of different size ions of thiocyanate, sulphite and ferricyanide [10].

The most frequently used methods for kaolinite thin films deposition are solution cast technique [11], spin coating and electrochemical techniques [12]. O. G. Abdullah et al. [11] used solution cast technique for preparing composite material light films with different ratio of polyvinyl alcohol/kaolinite, and thicknesses ranging between 0.20 mm and 0.44 mm, aiming to study their optical properties. Kaolin light addition leads to a decrease of the optical energy gaps, while the Urbach energy tends to increase. N. Kouider et al. [12] describes the growth of porous and corrosion resistant films, via electrochemical coating method, using kaolin on stainless steel surfaces as modified electrodes suitable for methanol or ethanol fuel cells.

For the sensor industry, the aim is to obtain thin films with oriented structure and a high active area. Nowadays, the pulsed laser deposition systems are widely used in laboratories having the potential to deposit various oxide-based materials as metallic oxides films with a high degree of adhesion onto substrate [13,14]. The experimental set-up of the PLD system is simple and versatile with great development potential and compatibility for a wide variation of oxides and hydroxides. By using pulsed lasers and a vacuum chamber, a variety of stoichiometric oxide films can be grown in vacuum, reactive or inert background gas without the need for further processing [15]. Based on the vast applications (i.e., biotechnology, microelectronics, optoelectronics) that are relying on these oxides and hydroxides, an increasing research interest was observed regarding PLD technique in the processing of these materials. This laser-based method has many advantages, for example low processing duration for thin films of hundreds of nanometers and it is a non-polluting method, the laser being the energy source [16]. Moreover, PLD can produce films with excellent adhesion, due to the high energy of the species reaching the substrate [17,18]. These unique features of laser-based methods are suitable for the deposition/transfer of kaolinite thin films in sensor-based applications. Our work is focused on producing for the first time, via laser-based techniques, thin films of kaolinite with well controlled structure and aims to overcome the limitations of the classical deposition methods (cost, processing time, adhesion). In our experiments the laser methods bring the advantage of good thickness control. The ability of the laser methods to grow oriented and stoichiometric films of lamellar layered double hydroxides at room temperature has been previously demonstrated [19,20].

## 2. Materials and Methods

### 2.1. Materials

**Figure 1:**
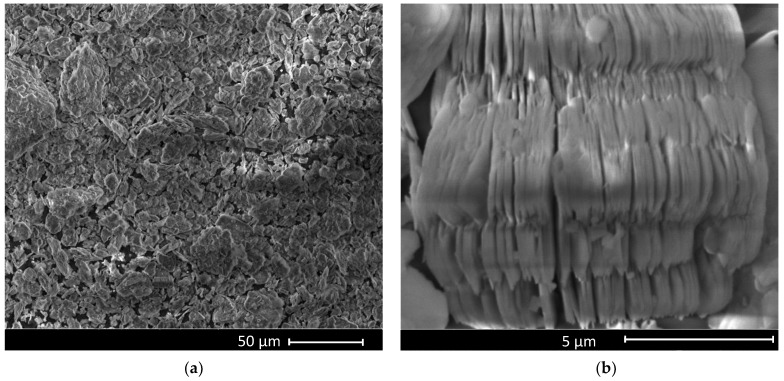
SEM images representing the topography of kaolinite powder with the magnifications of 1000× (**a**) and 20000× (**b**).

The following product was purchased and used without further purification: Kaolinite (KAOLINITE, NATURAL) FLUKA 03584-250G from (SIGMA-ALDRICH, CHEMIE GmbH, Riedstrt. 2 D-89555 Steinheim, Germany). The SEM images presented in Figure 1 highlight the lamellar structure of kaolinite powder. This clay powder was used in the preparation of PLD targets (by pressing with a hydraulic press with a pressure of (≈20 MPa) applied to our kaolinite material) and MAPLE (by using an aqueous solution with 5 or 10 wt.% kaolinite in deionized water (as matrix)).

### 2.2. Lamellar Thin Films Deposition

Pulsed laser deposition (PLD) and matrix-assisted pulsed laser evaporation (MAPLE) experiments were performed starting from pressed kaolinite powder as targets for PLD technique, or from water-dispersed kaolinite solutions (5 or 10 wt.%) and then solidified/frozen by using liquid nitrogen, for the MAPLE method.

For PLD depositions, a Nd:YAG pulsed laser (Continuum Surelite II & Neocera workstation, Santa Clara, CA, USA) working in IR, VIS or UV and an ArF excimer laser at 193 nm were used to irradiate the target while for MAPLE, the fourth harmonic of the Nd:YAG pulsed laser working at 266 nm wavelength was chosen, based on our previous experiments [21,22,23,24,25,26]. The repetition rate was set at 10 Hz, the target and the substrate (Si <110>) were kept parallel at 4 cm distance, with the laser beam translated and the target rotated during depositions, for both PLD and MAPLE experiments. Kaolinite thin films were deposited by PLD method, as a result of 20.000–40.000 pulses at laser fluences ranging from 1 up to 5 J/cm^2^, while for MAPLE technique, 72.000 pulses were used to irradiate the target with a laser fluence between 1 and 2 J/cm^2^. The films deposition was made in vacuum conditions (1 × 10^−5^ mbar), with a small increase of the pressure during irradiation. For both PLD and MAPLE during experiments, the substrate (Si) was kept at room temperature (RT).

### 2.3. Characterization

X-ray diffraction (XRD) (PANalytical X’Pert MPD system, Almelo, The Netherlands) with a wavelength of 0.15418 nm was used for the crystalline structure investigation, for both powder material/target and deposited thin films. The HighScore software package (Version 4.0, PANalytical B.V., Almelo, The Netherlands, 2013) was used for the structural data analysis. X-ray photoelectron spectroscopy (XPS) survey spectra and high-resolution XPS scan spectra were acquired using an Escalab Xi+ system (Thermo Scientific, Waltham, MA, USA).

Thin films morphology and topography were analyzed by atomic force microscopy in ambient conditions, in non-contact mode-AFM XE-100 type from Park Systems, (Suwon, South Korea) and by scanning electron microscopy-SEM (FEI, model Inspect S50, Hillsboro, OR, USA) at an accelerating voltage of 10 kV.

In order to study the wettability of the surfaces as well as to determine the total free energy of the surface (SFE), contact angle measurements (WCA) were performed. For this matter two types of liquids were used: water (double distilled) and methylene iodide (MI), water as polar liquid and MI as nonpolar liquid. Kaolinite thin film wettability was analyzed by sessile drop method using static contact angle measurements. The WCA measurements were made using an optical Contact Angle Tensiometer, CAM 101, from (KSV Instruments Ltd., Espoo, Finland), equipped with a CCD camera, an LED source, stands for substrate and a standard syringe from Hamilton (1000 µL). These wetting experiments were performed in the atmospheric environment at room temperature (RT) by placing the liquid droplet with the volume of 2 µL on the investigated surface. To calculate the total surface free energy (SFE) of the solids the Owens, Wendt, Rabel, and Kaelble (OWRK) [27,28] method was used. The solvents used in this SFE study for wetting experiments were: bidistilled water and methylene iodide (MI). The values for these solvents in terms of their dispersive and polar components are given in Table 1. These values used in the calculation of SFE are taken from the database of the device used to measure the contact angle.

The DRIFT spectra of as deposited PLD films were collected using a JASCO (Tokyo, Japan) FT/IR-4700 spectrometer with a diffuse reflectance accessory (PIKE). The Si(001) substrate spectrum was extracted from the acquired spectra of the films.

## 3. Results and Discussion

**Figure 2:**
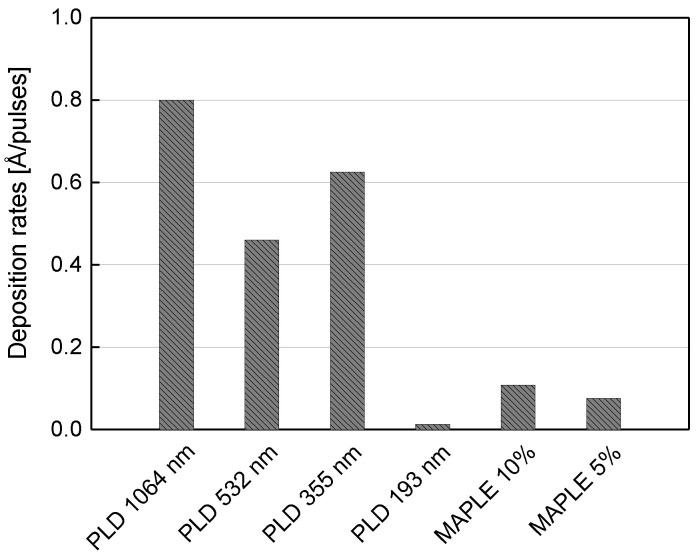
Kaolinite thin films deposition rates. Comparison between deposition methods: PLD and MAPLE.

Taking into account the profilometric measurements, it was observed that kaolinite thin films deposited as a result of target irradiation with 40.000 pulses for PLD and 72.000 pulses for MAPLE have thicknesses between tens of nanometers and a few microns, depending on the laser wavelength used for experiments. This leads to high deposition rates for PLD experiments, excepting 193 nm deposition and, considerably lower rates for MAPLE depositions. The comparison between deposition rate of kaolinite thin films using PLD and MAPLE methods is presented in Figure 2.

**Figure 3:**
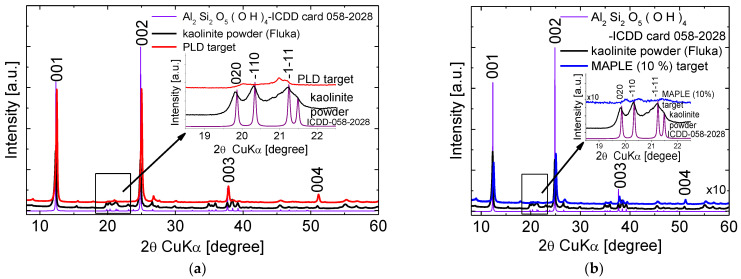
XRD patterns of the kaolinite commercial powder, (**a**) PLD target and (**b**) MAPLE target.

The commercial powder from Fluka contains as dominant phase anorthic kaolinite-1A Al_2_ Si_2_O_5_(OH)_4_ (ICDD card 00-058-2028) and traces of monoclinic-(SiO_2_)_×_ (ICDD card 00-042-005). The same kaolinite 1A phase is found in the targets. The particular preparation of the PLD target, by pressing, induced increased intensities of the (00*l*) reflections marking a preferred orientation of the layers along the *c*-axis (Figure 3a) The MAPLE target prepared by dispersing the kaolinite powder in water which is then dried in ambient atmosphere, exhibits also a preferred orientation along the *c*-axis due to the restacking of the layers in the aqueous solution (Figure 3b).

There is a large number of studies on the structure and proper definition of order and disorder in clays crystal structures [29]. The term of crystallinity is to be rather avoided in connection with clays and other parameters were introduced to describe the variety of order or disorder forms occurring in phyllosilicates [30]. We calculated two of such order-disorder indexes: the Hinckley index (HI), the most widely used, allowing a semi-quantitative evaluation of the degree of order-disorder of the kaolinite lattice, which was proposed in 1963 [31] and the Aparacio-Galan-Ferrel index (AGFI) proposed in 2006 [32]. In both cases, the indexes are determined by using reflections in the range 19° to 23° 2*θ*, considered to be sensitive to the structural defects as random and interlayer displacements. The HI includes the background in the calculation while, AGFI only the peaks intensities, which allows a higher degree of confidence. Values of HI < 0.5 and AGFI < 0.90 designates a disordered kaolinite while HI > 1.5 and AGFI >1.6 [29,30] characterizes an ordered kaolinite. FWHM-001 (F1) and FWHM-002 (F2) indexes determined as the width at half height of the basal reflections of kaolinite, (001) and (002), are the only one derived from oriented aggregates. Values (in degree) range from >0.4 (disordered) to <0.3 (ordered) [29]. The crystallite size D_001_ obtained from FWHM-001 through the Scherrer formula describes the thickness of coherent diffraction domain along the *c*-axis, which is the stacking axis of platy phyllosilicate layers. Awad et al. [33] found correlations between the crystallite size D_001_ of 28 kaolinite samples and their HI order-disordered index.

Table 2 presents the structural data and the above-described indexes for the commercial powder and the prepared targets. The *d*_001_ basal spacing calculated via the Bragg equation was also included. The targets preserved almost the same crystallographic parameters, namely the reduced unit cell parameters, hence their unit cell volume, as the commercial powder. The data shows that the commercial powder according to its HI, AGFI, F1 and F2 indexes is a low-defect kaolinite. Its crystallographic phase composition with very low amount of impurities and its high purity chemical composition, as the XPS data presented later, explain this result. The PLD target shows an increase of all the calculated order-disorder indexes. A. La Iglesia reported that very high static pressure induced disorder in kaolinites such as fractures, deformation and rolling of the layers [34,35]. The pressure of ≈ 20 MPa for the manufacture of the target/pellet used in the PLD experiments is 3 orders of magnitude less as those reported by La Iglesia. Actually, at this low-pressure value an increase of the coherence stacking layers distance occurs. The MAPLE target presents a very slight decrease of the D_001_ size value and of the AGFI index as a result of the dissolution in water and the presence between the kaolinite layers of a larger number of polar hydroxyl groups.

There is a linear relationship between the overall order-disorder index AGFI and the size of the coherent diffraction domain perpendicular to the (001) plane, D_001._ In conclusion the XRD analysis evidenced that the targets preparation procedures preserved all the intimate structural features of the commercial powder used for their preparation.

The XPS investigation employed to identify the chemical composition of lead to a ratio Al/Si of 0.82 (Table 3). The larger proportion of Si is consistent with the XRD observation of a small amount of SiO_2_.

**Figure 4:**
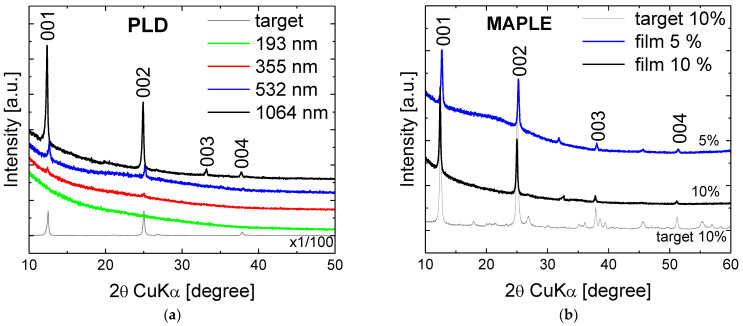
XRD patterns of the films deposited by PLD (**a**) and MAPLE (**b**) and their corresponding targets.

The PLD films show reflections except for the λ = 193 nm (Figure 4a and Table 4). No (hkl) with *h* ≠ 0 and *k* ≠ 0 is observable. For λ = 1064 nm all the four (00*l*) peaks are visible, and the intensities are high which turns this wavelength as the most effective one. The F1 and F2 values are <0.3° indicative of ordered kaolinite. In addition, the coherent domain size values characterizing the extension of the staking of layers, D_001,_ are slightly larger for the 1064 nm and 532 nm than the corresponding value for PLD target, indicative of highly oriented films. The *d*_001_ basal spacing values are similar to their corresponding PLD target, with a slight increment for the 1064 nm film, marking slight larger interlayer distance.

In the XRD patterns of MAPLE films (Figure 4b and Table 4) all the four (00*l*) reflections are clearly visible even for the film originating for the target with a lower concentration of 5%. The F1 and F2 indexes are also <0.3° with coherent domain sizes characterizing ordered kaolinite samples. The *d*_001_ basal spacing values are consistent with *d*_001_ spacing of the MAPLE (10%) target.

In conclusion the transfer of kaolinite as films via laser techniques from properly prepared targets produced highly oriented and ordered films in particular for 1064 nm wavelength for PLD and 10% concentration for MAPLE.

Scanning electron microscopy (SEM) and atomic force microscopy (AFM) used to study the surface morphology and the topography of the samples show clay films with a compact appearance, completely covering the substrate, with three-dimensional clusters and high roughness, especially for higher wavelengths (532 nm, 1064 nm). The decrease in laser fluence leads to a decrease in film roughness and a change in surface microstructure appearance. At 193 nm wavelength, the film surface is structured, dense, presenting grains in the range of tens of nanometers.

**Figure 5:**
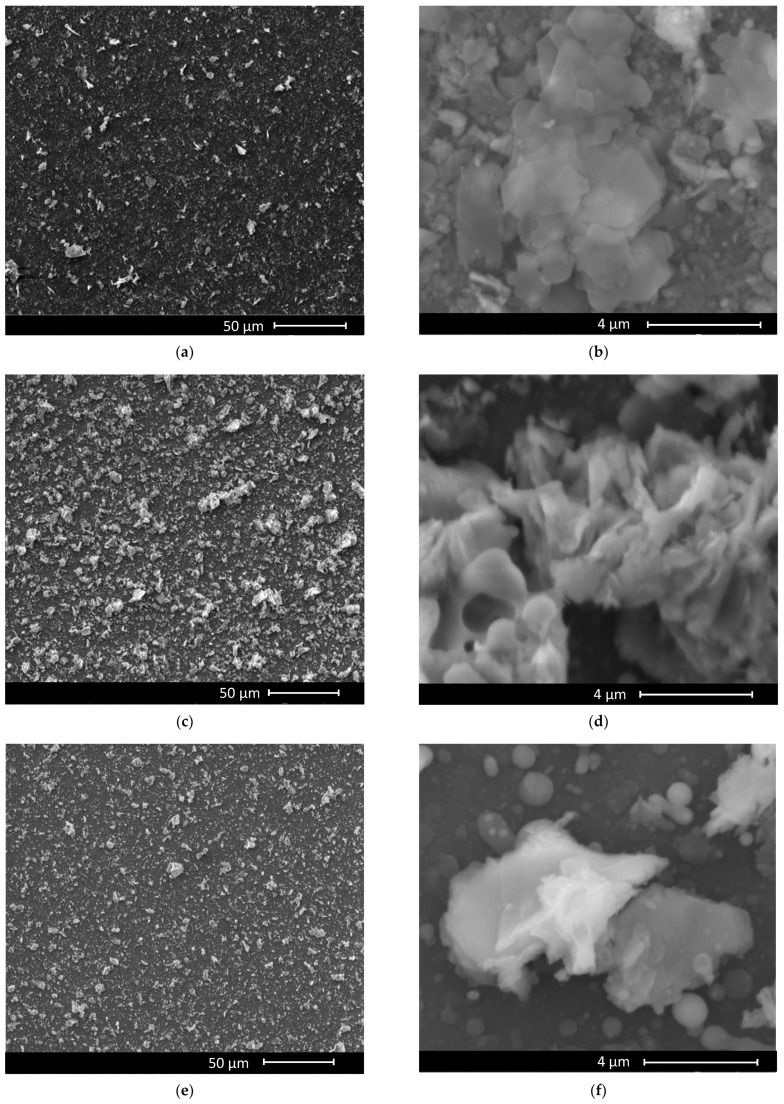

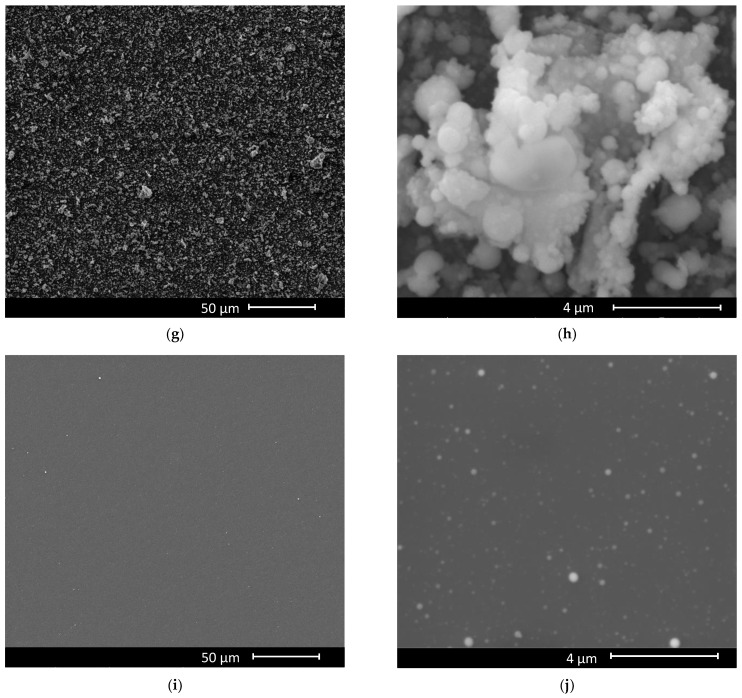
Grown films SEM images of MAPLE (**a**,**b**), PLD 1064 (**c**,**d**) and PLD 532 nm (**e**,**f**).Grown films SEM images of PLD 355 nm (**g**,**h**) and PLD 193 nm (**i**,**j**).

**Figure 6:**
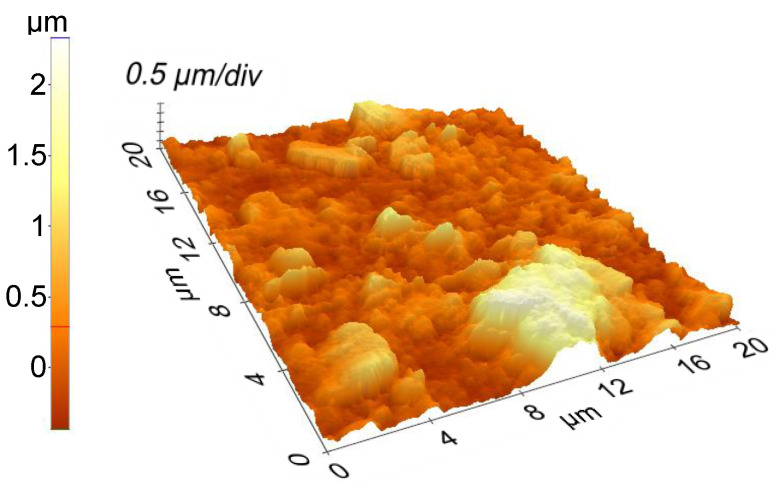

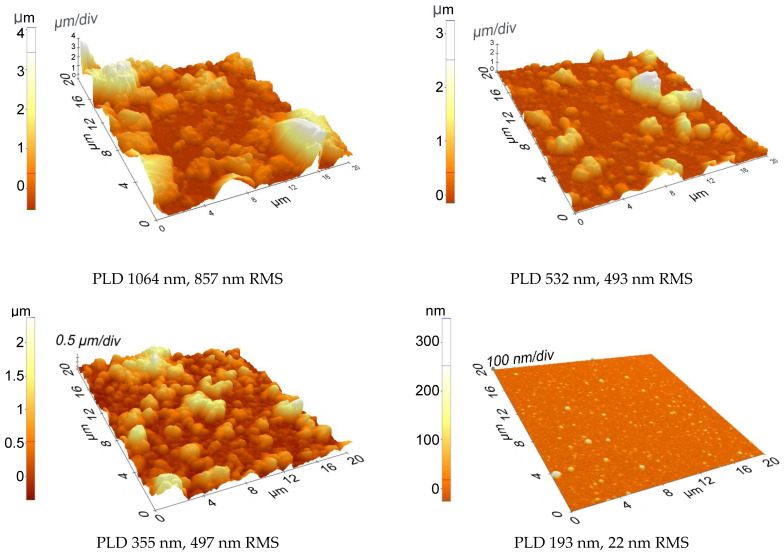
AFM image of films deposited by MAPLE; RMS roughness is 486 nm. AFM images and RMS roughness of films deposited by PLD.

**Figure 7:**
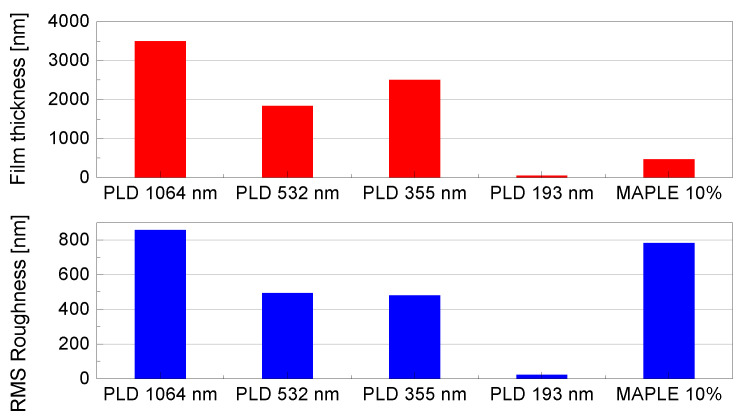
RMS roughness and thickness of films deposited by MAPLE and PLD.

Figure 5 displays the SEM, whereas Figure 6 shows AFM images and the RMS roughness depending on the wavelength used for the experiments, correlated with the film thickness (Figure 7).

**Figure 8:**
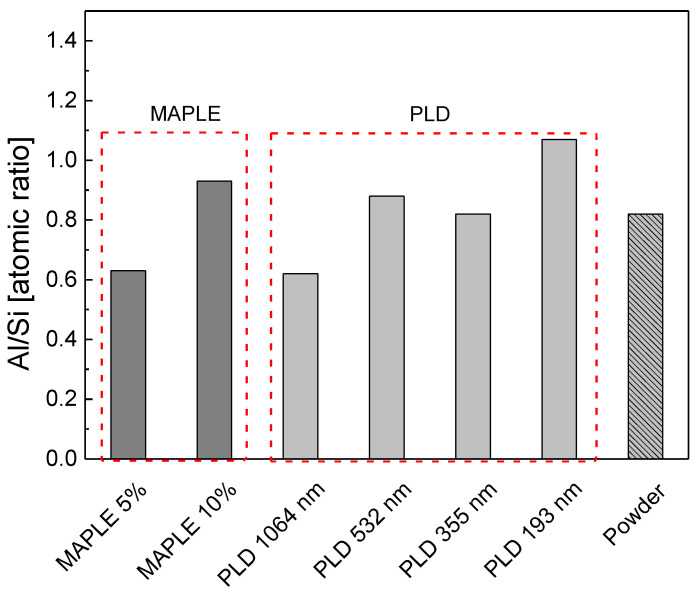
Al/Si atomic ratio for films deposited by MAPLE and PLD, as resulted from XPS survey.

Samples elemental composition as analyzed by XPS is presented in Table 5 and Figure 8 it displays the presence of Si, Al and O, with similar composition found in the starting material, and ratio Al/Si smaller than 1, for most of the samples. The 193 nm PLD deposition is accompanied by Si leaching, consistent with the XRD measurements, supporting the decomposition of kaolinite structure.

**Figure 9:**
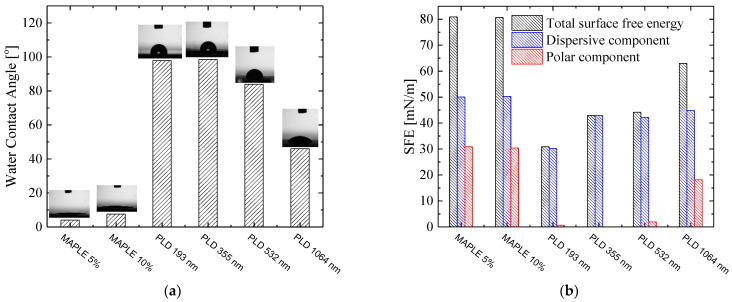
Contact angle (**a**) and surface energy components (**b**) for films deposited by MAPLE and PLD.

In Figure 9a are shown water contact angle measurements (WCA) for kaolinite films obtained by PLD and MAPLE.

In general, the wettability of surfaces and implicitly the measurement of the water contact angle depends on many factors. For instance, in the case for clay minerals, wettability of the surface and the water contact angle is more complicated and difficult to determine and can be affected by: experimental procedure, film deposition method, deposition parameters, surface roughness, humidity, temperature, heterogeneity of surface, adsorption phenomena and particle size [36]. Some authors have reported that pure kaolinite has a hydrophilic or moderate hydrophilic character due to the hydroxyl groups on the surface [36,37,38,39,40] having a water contact angle value of 17–26°, respectively, 46.1° and 42°. In addition, in [36,41] the wetting characteristics of the silica tetrahedral face and alumina octahedral face of kaolinite was studied. The result of these study shows that the silica face of kaolinite has a modest level of hydrophobicity and the alumina face of kaolinite is hydrophilic.

In our case, similar values to those in the literature were found for water contact angle, namely 46° and was obtain for kaolinite film deposition by IR PLD techniques. As can be seen from the graph, MAPLE leads to superhydrophilic films with contact angles in the range 4° and, respectively, 8°, depending on both the microstructure and surface roughness at micro and nano scale. Hydrophilic films with a water contact angle of about 46° can be obtained for IR PLD. In the case of PLD, at lower wavelengths it leads to water contact angles of around 98°.

For kaolinite films, surface energy and its components (polar and dispersive) were calculated by measuring the contact angle with water and methylene iodide. The surface energy and the contribution of polar and dispersive component are shown in the graph from Figure 9b. For the films deposited by MAPLE method, the polar components of surface energy are around 30 mN/m. The surface energy of the kaolinite films deposited by PLD was approximately 30.86 mN/m for wavelengths of 193 nm, 42.9 mN/m for 355 nm and 44.14 mN/m for 532 nm. The most important contribution is due to the dispersive components. For wavelength of 1064 nm, the surface energy of kaolinite film was 62.97 mN/m, polar component having the value of 18.16 mN/m.

From the results presented in this paper it is observed that the kaolinite films deposited by MAPLE technique have a higher affinity for water while the films deposited by PLD at lower wavelengths show a moderately water-repellent character. Kaolinite films deposited by IR PLD show moderate hydrophilicity.

These wetting behaviors are important and make them suitable for various applications in which these kaolinite films deposited by PLD and MAPLE can be used as active surfaces by functionalization or grafting with hydrophilic groups, as a support for use such as a sensor for protein detection, or as composite materials for temperature sensor and food packaging including.

**Figure 10:**
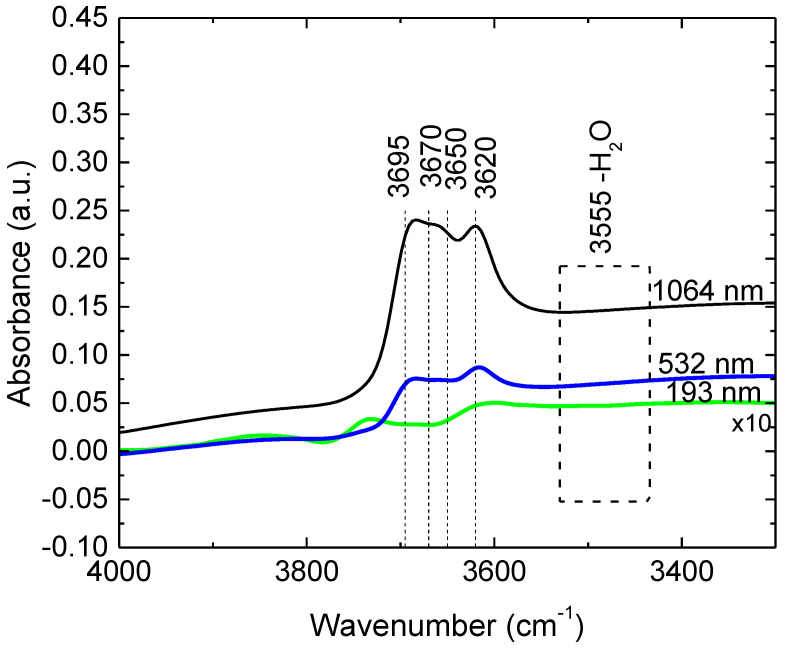
DRIFT spectra of the hydroxyl stretching region of the films deposited via PLD at 193 nm, 532 nm and 1064 nm.

The structural transformation and the reactivity of kaolinite involve dehydroxylation reactions [42]. In order to survey the effect of laser interaction on the molecular structure and in particular on the potential degradation of the hydroxyl groups in kaolinite we compared the DRIFT spectra of the PLD films deposited at 193 nm, 532 nm and 1064 nm, respectively. In the OH stretching region has four infrared active modes centered at 3695, 3670, 3650 and 3620 cm^−1^. According to Farmer [43] the band at 3695 cm^−1^ is due to the in-phase inner surface hydroxyl stretching vibration, the two at 3670 and 3650 cm^−1^ to the out-of–phase vibrations of the inner surface hydroxyl and the band at 3620 cm^−1^ is the hydroxyl stretching vibration of the inner hydroxyl. The inner surface hydroxyls are also called outer hydroxyl groups and are situated in the outer upper, unshared plane, whereas the inner hydroxyl groups are located in the plane share with the apical oxygens of the tetrahedral sheet [44]. The resolution and the intensity of these bands depend on the defects in the kaolinite. However, the assignment of the hydroxyl stretching bands was and still is under constant review [45,46,47]. Thus, the information gained through such systems as *c*-oriented kaolinite films will add more knowledge on kaolinites and their behavior as functional material in prospects of future applications. The DRIFT spectra of the hydroxyl stretching region of the films deposited via PLD are presented in Figure 10. The spectrum of the film deposited at 193 nm was multiplied by 10 in order to be visualized. As in can be see the 3620 cm^−1^ peak corresponding to the inner hydroxyl groups are almost not shifted for the 532 nm and 1064 nm films (positioned at 3616 cm^−1^ for 532 nm film and at 3617cm^−1^ for 1064 nm film, respectively). The band is shifted to 3609 nm for 193 nm film signing structural alteration. Similarly, the in-phase inner-surface band at 3695 nm appears at 3693 cm^−1^ for 1064 cm^−1^ and at 3694 cm for 532 nm film while its position for the film deposited at 193 nm is seriously shifted towards 3730 cm^−1^. The two bands corresponding the readily accessible out-of-phase outer hydroxyl groups are not so discernable and almost vanish for the 193 nm film, marking for this film a partial dexydroxylation accompanied by its structural deterioration. The spectra allure are similar to those of high-defect kaolinites [48], to kaolinites subject to mechanochemical activation via grinding which will decrease their crystallites sizes [48,49,50] or/and subjected to thermal treatments [42]. That means that all that the vibration of the inner surface hydroxyl groups are free and the OH groups are more readily accessible for exchange increasing their potentiality for functionalization. The DRIFT data are consistent with the XRD results indicating the 1064 nm as the conducting to the most “crystalline” film while the film deposited at 193 nm is amorphous to XRD. For all these films deposited via PLD no band around 3555 cm^−1^ ascribed to water [51] is observable. The result is consistent with hydrophobicity of the PLD films of the as revealed by contact angle measurements. It is to be mentioned that the 1064 nm film exhibit a lower hydrophobicity probably due to its high roughness.

## 4. Conclusions

We have successfully synthesized crystalline thin films of kaolinite using laser techniques, PLD and MAPLE, preserving the structure and chemical composition of the initial raw kaolinite. All the films are highly oriented along the *c*-axis. The 1064 nm wavelength for PLD secures the production of a thick and highly textured film while at λ = 193 nm an extremely thin and amorphous to XRD film is obtained. MAPLE proves to be effective for the deposition of highly oriented kaolinite films even at 5 wt.%. kaolinite in the target. It is a suitable technique for the deposition of clay films with pronounced hydrophilic character. To conclude our results, kaolinite thin films can be further used as active surfaces in electrochemical sensing.

## Figures and Tables

**Table 1:** Total surface energy, dispersive and polar components for the solvents.

Solvent	Total Surface Energy [mN/m]	Dispersive Component [mN/m]	Polar Component [mN/m]
Bidistilled water	72.80	21.80	51.00
Methylene iodide	50.80	50.80	0.00

**Table 2:** Structural data of the commercial powder and the targets obtained from XRD analysis.

Sample	Reduced Unit Cell Parameters							d_001_ (Å)	HI	AGFI	F1 (deg)	F2 (deg)	D_001_ (nm)
	a (Å)	b (Å)	c (Å)	α (°)	β (°)	γ (°)	Vol(Å^3^)						
ICDD card 00-058-2028	5.156	7.409	8.950	88.18	89.81	75.34	330.59	7.1697					
powder	5.14(8)	7.40(9)	8.95(8)	88.0514(3)	89.91(2)	74.94(2)	328.67	7.214	1.14	1.53	0.27	0.23	33.7
PLD target	5.15(7)	7.38(6)	8.94(9)	88.0055(3)	89.62(2)	74.91(2)	328.14	7.102	1.68	1.63	0.24	0.21	37.4
MAPLE (10%) target	5.16(8)	7.36(7)	8.9(1)	88.9991(3)	89.61(2)	75.09(1)	327.80	7.081	1.25	1.04	0.30	0.27	31.0

**Table 3:** Atomic composition for kaolinite powder.

Element	Atomic Ratio %
O1s	59.88
Si2p	19.15
Al2p	15.72
C1s	5.25

**Table 4:** Structural data of the films deposited via PLD and MAPLE.

Films	d_001_ (Å)	F1(deg)	F2(deg)	D_001_ (nm)
MAPLE 5%	7.014	0.274	0.247	33.8
MAPLE 10%	7.126	0.213	0.210	39.2
PLD 1064 nm	7.172	0.217	0.212	38.6
PLD 532 nm	7.005	0.197	0.184	42.4
PLD355 nm	7.127	0.243	0.172	34.4

**Table 5:** Films atomic composition and Al/Si ratio.

Atomic %	MAPLE 5%	MAPLE 10%	PLD 1064 nm	PLD 532 nm	PLD 335 nm	PLD 193 nm
O1s	66.22	65.33	62.79	60.92	62.63	63.06
Si2p	20.73	17.95	22.97	20.69	20.51	17.80
Al2p	13.05	16.71	14.24	18.39	16.85	19.13
Al/Si	0.63	0.93	0.62	0.88	0.82	1.07

## Data Availability

Not applicable.
